# Consistent Differences in Field Leaf Water-Use Efficiency among Soybean Cultivars

**DOI:** 10.3390/plants8050123

**Published:** 2019-05-10

**Authors:** James Bunce

**Affiliations:** USDA-ARS Adaptive Cropping Systems Lab (Retired), Beltsville, MD 20705, USA; buncejames49@gmail.com; Tel.: +1-410-271-8177

**Keywords:** water use efficiency, soybean, stomatal conductance, photosynthesis, carbon isotope

## Abstract

High intrinsic water-use efficiency (WUE_i_), the ratio of leaf photosynthesis to stomatal conductance, may be a useful trait in adapting crops to water-limited environments. In soybean, cultivar differences in stomatal response to vapor pressure deficit have not consistently translated into differences in WUE_i_ in the field. In this study, six cultivars of soybeans previously shown to differ in WUE_i_ in indoor experiments were grown in the field in Beltsville, Maryland, and tested for mid-day WUE_i_ on nine clear days during the mid-seasons of two years. Measurement dates were chosen for diverse temperatures, and air temperatures ranged from 21 to 34 °C on the different dates. Air saturation deficits for water vapor ranged from 0.9 to 2.2 kPa. Corrected carbon isotope delta values for ^13^C (CID) were determined on mature, upper canopy leaves harvested during early pod filling each year. WUE_i_ differed among cultivars in both years and the differences were consistent across measurement dates. Correlations between mean WUE_i_ and CID were not significant in either year. It is concluded that consistent cultivar differences in WUE_i_ exist in these soybean cultivars under field conditions, but that carbon isotope ratios may not be useful in identifying them because of cultivar differences in mesophyll conductance.

## 1. Introduction

With projected increased frequency of drought, and decreased availability or increased cost of water for agriculture, increasing the efficiency of water use in agriculture is an important goal [1]. In addition to crop management strategies, inherent increases in crop water-use efficiency (WUE) could be useful in reaching this goal. For a given leaf to air difference in water vapor pressure (VPD), the ratio of photosynthesis to transpiration, termed leaf water-use efficiency, is inversely related to the ratio of substomatal CO_2_ concentration to ambient CO_2_ concentration (C_i_/C_a_) [1]. The realization that the discrimination between isotopes of carbon in CO_2_ in leaf photosynthetic CO_2_ fixation was related to C_i_/C_a_ [2] led to many tests of intraspecific relationships between corrected isotope delta values for ^13^C (CID) and crop WUE. Significant correlations between CID and crop WUE have been found in many crop species, such as wheat [3], peanut [4], tomato [5,6], cowpea [7], cotton [8], barley [9], and sugar beet [10]. However, partly because of correlations between crop WUE and leaf size, plant size, and leaf CO_2_ assimilation rate in some species, improved crop WUE has been no guarantee of increased yield in dry conditions [1,11]. Clearly, other plant variables must also be managed. Sinclair [12] has argued that stomatal response properties limiting transpiration at high VPD, which would increase WUE, would have yield benefits in many agricultural species and environments.

In spite of common correlations between CID and crop WUE, in some cases CID has not been correlated with leaf gas exchange measurements of C_i_/C_a_ [5,13,14]. This type of result is of concern for the general usefulness of CID as a selection tool to change C_i_/C_a_. Warren et al. [15] argued that mesophyll conductance (g_m_) to CO_2_ movement from the sub-stomatal air space to the site of fixation inside the chloroplast varied enough among species to disrupt relationships between CID and C_i_/C_a_. Barbour et al. [16] argued that variation in g_m_ in barley disrupted correlations between CID and WUE, as did Gioliani [13] in rice. Seibt et al. [17] also emphasized that CID was not directly related to the C_i_/C_a_ ratio, but to the C_c_/C_a_ ratio, where C_c_ is the CO_2_ concentration at the site of fixation inside the chloroplast. Easlon et al. [18] provided evidence of the importance of genetic variation in g_m_ to CID in *Arabidopsis thaliana*. Because g_m_ may vary with temperature [19,20], light [21] and C_i_ [22], it is to be expected that CID may not always correlate highly with leaf C_i_/C_a_.

Regardless of variation in g_m_, leaf WUE at a given VPD would be proportional to C_i_/C_a_ [23]. The ratio C_i_/C_a_ depends on the ratio of photosynthesis to stomatal conductance, which is termed intrinsic leaf water-use efficiency (WUE_i_) [17]. While operational C_i_ is somewhat conservative in the steady-state over changes in light and temperature [24] it certainly varies with VPD in many cases. In soybeans, much prior work focused on “slow wilting” soybeans in which transpiration increased less rapidly with increasing VPD [25,26,27] as genetic resource to increase WUEi. However, it is disconcerting that genotypic differences in responses of transpiration to VPD in soybeans, identified in controlled environment tests and field tested in North Carolina [25,26,27] were not evident when tested in California [28]. In the tests in California, no genetic differences in WUE_i_ occurred. In this study, cultivars of soybean identified in tests in indoor controlled environment chambers as differing in C_i_/C_a_ and WUE_i_ at a single VPD were grown in the field in Beltsville, Maryland, over two years to test whether this method of identification of high WUE_i_ lines produced consistent differences in WUE_i_ over a range of temperature and VPD conditions in the field. Leaf gas exchange was measured on nine clear days in mid-summer, chosen to have a wide range of air temperature and VPD values. Cultivars were compared for steady-state values of WUE_i_ to determine whether any cultivar differences in WUE_i_ were consistent across measurement days and years. Mature leaves harvested at early pod fill were analyzed for CID values for tests of correlations between CID and the mean leaf WUE_i_ of the cultivars.

## 2. Results

**Figure 1:**
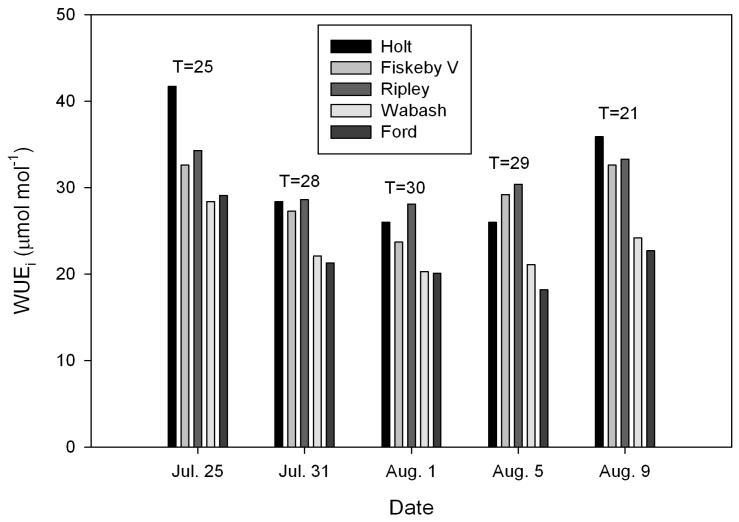
Intrinsic leaf water-use efficiency (WUE_i_) in five cultivars of soybeans measured on five dates in 2017. Air temperatures (°C) during the measurements on each date are provided.

**Figure 2:**
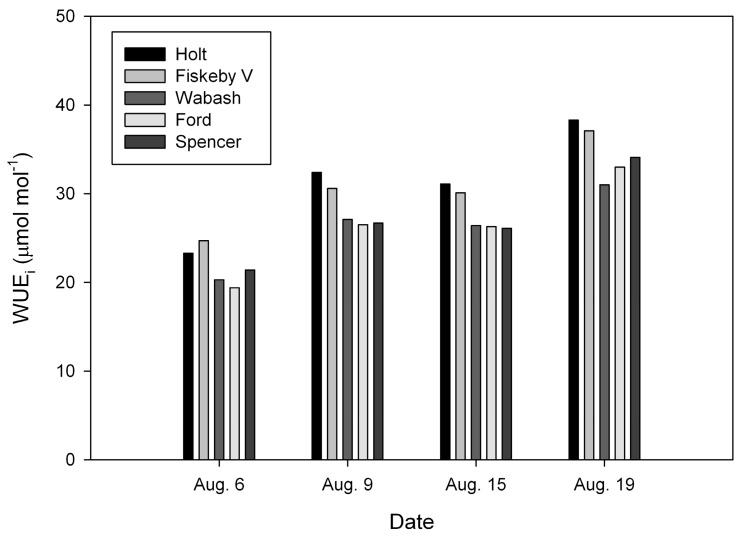
Intrinsic leaf water-use efficiency (WUE_i_) in five cultivars of soybeans measured on four dates in 2018. Air temperatures (°C) during the measurements on each date are provided.

Air and leaf temperatures during the leaf gas exchange measurements both ranged from 21 to 34 °C on the nine different dates (Figure 1 and Figure 2), and air saturation deficit (ASD) values ranged from 0.9 to 2.2 kPa. The correlation coefficient between ASD and air temperature was 0.399, which was not significant (*P* = 0.288).

The cultivar × date interaction term was significant for WUE_i_ in 2017 (Table 1), but was not significant in 2018 (Table 2), nor was it significant for A or g_s_ in either year. Despite the significant cultivar × date interaction for WUE_i_ in 2017, the cultivars were clearly divided into two consistent groups of cultivars with contrasting WUE_i_ on all of the measurement dates (Figure 1). Holt, Ripley and Fiskeby V all had higher WUE_i_ than did Ford and Wabash on each date. In 2017, the three cultivars with high WUE_i_ had both higher A and lower g_s_ than the two cultivars with low WUE_i_ (Table 3). In 2018, Holt and Fiskeby V again had higher WUE_i_ than Ford and Wabash, while Spencer had low WUE_i_, similar to Ford and Wabash (Figure 2). Relationships between absolute values of A, g_s_ and WUE_i_ were unclear in 2018, because Spencer had high A, but low WUE_i_, and Wabash, with low WUE_i_ also had low g_s_ (Table 4).

**Figure 3:**
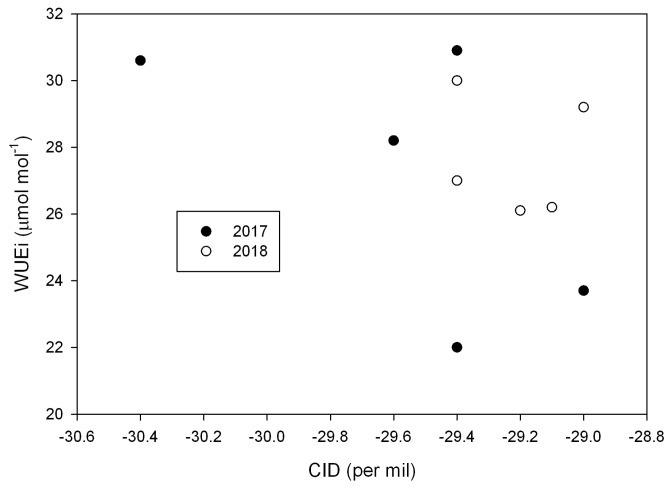
Relationships between mean intrinsic leaf water-use efficiency (WUE_i_) and corrected isotope delta values for ^13^C (CID) among five cultivars of soybeans in 2017 and 2018. Correlations between WUE_i_ and CID were non-significant at P = 0.05 in either year. See text for details.

Significant differences among cultivars in CID values occurred in both years, although differences were larger in 2017 than 2018 (Table 4). In 2017, Holt had a smaller (more negative) value than the other four cultivars. In 2018, Holt and Spencer had the smallest values. In neither year was there a significant correlation between mean WUE_i_ averaged over the measurement dates and CID (Figure 3). In 2017, the correlation coefficient was 0.647, with *P* = 0.238. In 2018, the correlation coefficient was 0.133, with *P* = 0.832.

On the two dates of each season of these experiments when mean leaf temperatures were close to those used in the previous indoor experiments, the correlation between C_i_ and C_c_ among cultivars was not significant in either year, with R^2^ = 0.194 (P = 0.458) in 2017, and R^2^ = 0.021 (*P* = 0.815) in 2018. The (non-significant) slopes were +0.622 in 2017, and −0.230 in 2018.

## 3. Discussion

It is highly likely that differences in g_m_ among the cultivars disrupted the overall correlations between WUE_i_ and CID, although this was specifically tested only on the two dates each year when leaf temperatures were similar to those in which g_m_ had been measured in prior indoor experiments. On those measurement dates, there were no significant correlations between C_i_ and C_c_ among the cultivars. Because CID is related to C_c_ rather than C_i_, cultivar differences in g_m_ could easily have caused the poor overall correlations between CID and C_i_ in this experiment. The larger intraspecific variation in g_m_ in rice of about 10× [13] than found in wheat, about 2× [29] could be related to the higher correlation between CID and WUEi in wheat [3] than in rice [13]. Measurements of g_m_ currently involve time-consuming leaf gas exchange procedures [30], so that measuring leaf WUE_i_ directly is probably more efficient than trying to correct CID values for g_m_ variation in order to estimate WUE_i_. Unlike a prior study of soybeans [31], correlations between g_s_ and g_m_ [23] were not strong enough to preclude differences in WUE_i_ in the cultivars examined here.

Although WUE_i_ varied substantially across measurement days, differences among cultivars were quite consistent across days and also over the two years of this study. These results suggest that WUE_i_ differences among these soybean cultivars were quite stable across a range of measurement temperatures and ASD, although maximum ASD values are not large in this environment. Any relationship between mean values of A or g_s_, and WUE_i_ suggested by the data for 2017 was disrupted in 2018. Cultivar differences in WUE_i_ among these soybean cultivars were not consistently associated with differences in mean values of either A or g_s_, but with operational C_i_ values. Reasons for cultivar differences in operational C_i_ are not known, but may be important for improvements in crop WUE.

## 4. Materials and Methods

In 2017, soybean cultivars Fiskeby V, Ford, Holt, Ripley, and Wabash were planted on 21 June at the South Farm of the Beltsville Agricultural Research Center. In 2018, the same cultivars were planted on 26 June in the same field, except that the cultivar Spencer was grown in place of Ripley. Seeds were obtained from the United States Department of Agriculture (USDA) soybean germplasm collection. These cultivars were chosen based on prior comparisons of their leaf gas exchange when grown indoors [23]. Fiskeby V, Holt, and Ripley had relatively high values of WUE_i_, and Ford, Wabash and Spencer had relatively low values of WUE_i_ [23] under the single measurement condition used in that study. The soil of the test site was a silt loam, with a water table at about 1.5 m depth, and with phosphorus and potassium contents adequate for soybeans according soil tests, and a pH of about 6.5. In these field tests, plants were grown in single row plots, one meter apart, and thinned after the emergence to 25 plants per meter of row. There were six replicate plots per cultivar, with each plot at least 2 m in length.

In 2017 leaf gas exchange was measured using a CIRAS-1 portable photosynthesis system (PP Systems, Amesbury MA). With that system, leaf and air temperatures are not controlled, but cuvette air temperature is designed to be very similar to outside air temperature by the use of large ventilated heat exchangers. In 2018, a CIRAS-3 portable system was used, and air temperature was controlled using Peltier units to be equal to that of outside air at the time measurements were begun. On each day, measurements were begun near midday and were completed in less than 60 minutes, so the outside air temperature changed little over the course of the measurements each day. Preliminary measurements were made each day to adjust the water content of the inlet air such that the air surrounding the leaves during gas exchange measurements had approximately the same water vapor pressure as outside air. In measurements with both instruments, the CO_2_ concentration in the reference air stream was controlled to be 400 µmol·mol^−1^, and the CO_2_ concentration in the air surrounding the leaves was 370 ± 5 µmol·mol^−1^. This mode of operation was chosen in order that steady-state rates of leaf gas exchange could be measured within one minute of enclosing leaves in the cuvettes. Tests showed that stomatal conductance did not change within a minute of changing the water vapor or carbon dioxide content of air surrounding leaves. Measurement dates were chosen for clear sky conditions, with a range of air temperatures, and also had a range of air saturation deficits for water vapor (ASD). Because of frequent precipitation, soil water content was not low enough to limit leaf gas exchange. During the leaf gas exchange measurements, the photosynthetically active radiation always exceeded 1500 µmol m^−2^·s^−1^ inside the cuvette.

On each measurement day, the steady-state CO_2_ assimilation rate (A), stomatal conductance (g_s_), and sub-stomatal CO_2_ concentration (C_i_) were obtained on a single leaf of each of six replicate plots of each cultivar, in random order. Leaves chosen for measurement were fully expanded upper canopy leaves which were in full sunlight several minutes before enclosing in the leaf cuvette. Air saturation deficits for water vapor were calculated from the temperature and water vapor content of outside air just prior to the leaf gas exchange measurements.

In 2017, there were five measurement dates, from 25 July to 9 August, and in 2018, there were four measurement dates, from 6 August to 23 August. On the earliest measurement date each year, plant development ranged from late vegetative to early flowering stage, depending upon the cultivar, and on the last date, plants were in early to mid-pod filling stages, depending upon the cultivar. A few days after the last leaf gas exchange measurements each year, the terminal leaflet of a mature upper canopy leaf was collected from each replicate plot for all cultivars and freeze-dried for the determination of corrected isotope delta (CID) values for ^13^C. CID was determined on each leaf sample by the Cornell Isotope Laboratory.

The g_m_ of each cultivar previously measured in indoor experiments was used to calculate C_c_ values for leaf gas exchange measured in the field, in order to test the correlation between C_i_ and C_c_ across cultivars under field conditions. These calculations were made for a single date each year when leaf temperatures in the field were closest to those used to measure g_m_ in the indoor experiments, which was 25 °C [23]. The two dates were 31 July 2017, when leaf temperatures averaged 26.7 °C, and 15 August 2018, when leaf temperatures averaged 26.0 °C.

Two-way analysis of variance (ANOVA) was conducted to test for effects of cultivar, measurement date, and their interaction on A, g_s_, and WUE_i_. These tests were undertaken separately each year, because the cultivars tested differed between years, as did the measurement instruments. One-way ANOVA was used to test for cultivar differences in CID each year. Correlations between cultivar means of WUE_i_ and CID, and between C_i_ and C_c_ were tested separately each year.

## Figures and Tables

**Table 1:** Analysis of variance for measurements of water-use efficiency (WUE_i_) of five soybean cultivars measured on five dates in 2017.

Source	DF (Degrees of Freedom)	Sum of Squares	Mean Square	F-Value	P-Value
Cultivar	4	1812	453	27.9	<0.0001
Date	4	1796	449	27.7	<0.0001
Cultivar × Date	16	525	32.8	2.03	0.0178
Residual	105	1701	16.2		

**Table 2:** Analysis of variance for measurements of WUE_i_ of five soybean cultivars measured on four dates in 2018.

Source	DF	Sum of Squares	Mean Square	F-Value	P-Value
Cultivar	4	335	83.8	7.04	<0.0001
Date	3	2318	773	65	<0.0001
Cultivar × Date	12	182	15.9	1.28	0.245
Residual	96	1141	11.9		

**Table 3:** Mean values of A, g_s_, WUE_i_, and carbon isotope delta (CID) values of five soybean cultivars measured on five dates in 2017. Values within columns followed by different letters were significantly different at *P* = 0.05, using a protected Least Significant Difference (LSD) test.

Cultivar	WUE_i_ (µmol·mol^−1^)	A (µmol·m^−2^·s^−1^)	g_s_ (mol·m^−2^·s^−1^)	CID (per·mil)
Fiskeby V	28.2 b	27.9 b	0.990 b	−29.4 b
Ford	22.0 c	25.3 c	1.149 a	−29.6 b
Holt	30.6 a	29.6 a	0.967 b	−30.4 a
Ripley	30.9 a	28.9 ab	0.936 b	−29.4 b
Wabash	23.7 c	24.7 c	1.043 ab	−29.0 b

**Table 4:** Mean values of A, g_s_, WUE_i_, and CID values of five soybean cultivars measured on four dates in 2018. Values within columns followed by different letters were significantly different at *P* = 0.05, using a protected LSD test.

Cultivar	WUE_i_ (µmol·mol^−1^)	A (µmol·m^−2^·s^−1^)	g_s_ (mol·m^−2^·s^−1^)	CID (per·mil)
Fiskeby V	29.2 a	36.2 b	1.24 bc	−29.0 b
Ford	26.1 b	31.6 c	1.21 cd	−29.2 ab
Holt	30.0 a	39.6 a	1.32 b	−29.4 a
Spencer	27.0 b	39.0 a	1.44 a	−29.4 a
Wabash	26.2 b	29.8 c	1.14 d	−29.1 b
